# Impact of acquisition area on deep-learning-based glaucoma detection in different plexuses in OCTA

**DOI:** 10.1038/s41598-024-71235-3

**Published:** 2024-09-02

**Authors:** Julia Schottenhamml, Tobias Würfl, Stefan Ploner, Lennart Husvogt, Robert Lämmer, Bettina Hohberger, Andreas Maier, Christian Mardin

**Affiliations:** 1 Department of Ophthalmology, Universitätsklinikum Erlangen, Friedrich-Alexander-Universität Erlangen-Nürnberg, Erlangen, Germany; 2 Pattern Recognition Lab, Friedrich-Alexander-Universität Erlangen-Nürnberg, Erlangen, Germany

**Keywords:** Image processing, Machine learning, Diagnostic markers

## Abstract

Glaucoma is a group of neurodegenerative diseases that can lead to irreversible blindness. Yet, the progression can be slowed down if diagnosed and treated early enough. Optical coherence tomography angiography (OCTA) can non-invasively provide valuable information about the retinal microcirculation that has shown to be correlated with the onset of the disease. The vessel density (VD) is the most commonly used biomarker to quantify this vascular information. However, different studies showed that there is a great impact of the acquisition area on the performance of the VD to distinguish between glaucoma patients and a healthy control group. It also seems that the separate capillary plexuses are differently affected by the disease and therefore also influence the results. So in this study we investigate the impact of the acquisition area (3 $\times$ 3 $\textrm{mm}$ macular scan, 6.44 $\times$ 6.4 $\textrm{mm}$ macular scan, 6 $\times$ 6 $\textrm{mm}$ optic nerve head (ONH) scan) and the different plexuses on the machine-learning-based distinction between glaucoma patients and healthy controls. The results yielded that the 6 $\times$ 6 $\textrm{mm}$ ONH show the best performance over all plexuses. Moreover the deep learning-based approach outperforms the VD as a biomarker on every acquisition area and plexus. In addition to that, it also performs better than traditional biomarkers obtained from the OCT scans that are used in the clinical routine for diagnosis and progression tracking of glaucoma. Consequently, OCTA scans of the ONH might be a useful addition to OCT when studying glaucoma.

## Introduction

Glaucoma is an entity of neurodegenerative diseases that is among the leading causes of irreversible blindness in the world^1^. However, if diagnosed and treated early enough the progression can be slowed down. There are typically three types to diagnose or track the progression of glaucoma: structural (e.g. through optical coherence tomography (OCT)), functional (e.g. through visual field testing) or physiological measurements (e.g. intraocular pressure measurements). Although the pathophysiology of glaucoma is not yet fully understood, a correlation between a decreased retinal blood flow and a diagnosis of glaucoma has been reported^2–5^.

The retinal microcirculation can be visualized by a relatively new extension of OCT, called OCT angiography (OCTA). In order to quantitatively measure the amount of perfusion in the retina, the vessel density (VD) is most often used as a biomarker. It can be computed from en face OCTA projections as the fraction of vessel pixels in the image.

OCTA has some advantages compared to the previously mentioned methods. One biomarker of OCT images is the thickness of the retinal nerve fibre layer (RNFL), which decreases with an onset of the disease. Though, there is the so called floor effect that describes the problem that in advanced stages of glaucoma the RNFL is already so thin that changes cannot be measured anymore (this is dependent on the OCT device used, but usually around 50–70% of the RNFL thickness in healthy controls)^6–8^. However, studies have shown that even at these late stages, when RNFL thickness is no longer meaningful, a decrease in the VD can still be observed^9,10^.

Visual field testing on the other hand is used to measure the visual function. In contrast to OCT and OCTA it requires a great amount of cooperation from the patient. Moreover, there already has to be done considerable damage to the retinal structure (estimates of at least 25–35% of the retinal ganglion cells in the RNFL) in order to be able to observe visual field abnormalities^11^.

In addition to that, OCTA is the only non-invasive option to visualize the retinal microcirculation on a capillary resolution. Consequently this method can provide new insights about the vascular component in the pathophysiology of glaucoma^12^.

There have been several publications investigating the discriminative power of OCTA via the VD for distinguishing glaucoma patients from a healthy control group. One key finding was, that the acquisition area has a major impact on the performance of this biomarker. Chen et al.^13^ observed in their study, that 6 $\times$ 6 $\textrm{mm}$ scans acquired at the macula show comparable results to scans that picture the peripapillary region. However, 3 $\times$ 3 $\textrm{mm}$ scans centered at the fovea performed worse than the two aforementioned acquisition areas^14–16^. One reason for this might be that the regions that were mostly affected by a reduced VD were the superotemporal and the inferotemporal sector which mostly lie just outside the 3 $\times$ 3 $\textrm{mm}$ area but inside the 6 $\times$ 6 $\textrm{mm}$ area^17,18^. Consequently, changes in the VD can be observed in the larger but not in the smaller scans. The second key finding is that the choice of the vascular plexus also significantly impacts the results that can be obtained using VD^19–22^. A plausible rational is therefore that the separate capillary plexuses are involved in the pathophysiology of glaucoma in different ways.

In a previous study, Schottenhamml et al.^23^ trained a convolutional neural network (CNN) on 3 $\times$ 3 $\textrm{mm}$ macular scans. The results were comparable to the ones obtained using VD reported in literature for the 6 $\times$ 6 $\textrm{mm}$ macular or peripapillary scans and outperformed the VD from 3 $\times$ 3 $\textrm{mm}$ scans. The latter is not surprising since CNNs have often been shown to outperform traditional biomarkers for medical imaging tasks. Later, Bowd et al.^24^ trained CNNs on 4.5 $\times$ 4.5 $\textrm{mm}$ peripapillary OCTA scans to also distinguish between eyes of patients with glaucoma and healthy controls. They also reported improved classification using CNNs compared to traditional OCT and OCTA biomarkers. However, both studies only considered a single acquisition area on a different patient cohort, so the results are not comparable.

Consequently in this study we investigate the impact of the acquisition area on the machine-learning-based glaucoma detection in the different plexuses.

## Methods and evaluation

In order to evaluate the different acquisition areas we will subsequently describe the data used in this study, followed by the design of our experiments.

### Data

In total, 219 eyes from 110 subjects (117 eyes from 59 patients with glaucoma and 102 eyes from 51 healthy control cohorts) were identified from the Erlanger Glaucoma Registry (Erlangen Glaucoma Registry, ISSN 2191-5008, CS-2011; NTC00494923). All subjects received a standardized ophthalmological examination including automated visual field testing, fundus photography and measurement of intraocular pressure (IOP) by Goldmann tonometry.

The patients in each group were selected based on the following inclusion criteria: the control cohort was defined as eyes showing no systemic disease with ophthalmological involvement or ophthalmological dysfunction neither having had any ophthalmic surgery. Glaucoma suspects were defined as having a normal visual field, and an elevated IOP (above 21 mmHg, ocular hypertension, OHT) or showed additive glaucomatous optic disc damage classified by Jonas et al.^25^ (preperimetric glaucoma). Glaucoma patients showed perimetric field defects and alterations of the optic nerve head according to Jonas et al.^25^. This group was further subdivided into patients having an elevated IOP (above 21 mmHg, open angle glaucoma, OAG)) and those not having an increased IOP (normal tension glaucoma). All glaucoma patients enroled in this study had bilateral glaucoma and showed signs of the disease in both eyes. In the glaucoma cohort are 20 eyes with OHT, 18 eyes with preperimetric glaucoma and 32 eyes of patients with normal tension glaucoma. The open angle glaucoma group consists of 35 eyes with primary open angle glaucoma and 12 eyes with secundary open angle glaucoma.

Exclusion criteria were an age below 18 years and any further eye disorders and/or systematic disorders with ocular involvement at the time of enrolement. All patients enrolled in this study were caucasian. The mean and standard deviation of the age distribution and refraction sphere for the healthy controls and the glaucoma patients and the perimetric data (mean deviation (MD) and square root of loss variance (sLV)) for the glaucoma patients is given in Table 1.

**Table 1:** Mean and standard deviation of the age distribution and refraction sphere for the healthy controls and the glaucoma patients and the perimetric data (mean deviation (MD) and square root of loss variance (sLV)) for the glaucoma patients.

	Healthy controls	Glaucoma patients
Age [years]	67.38 ± 15.08	61.56 ± 11.12
Refraction sphere [D]	− 1.25 ± 2.30	− 1.29 ± 2.24
MD [dB]		6.18 ± 6.68
sLV [dB]		5.01 ± 3.15

OCT and OCTA imaging was done using a SOLIX fullrange device (Optovue Inc, Fremont, California, USA). Images were acquired with a lateral resolution of 15 $\upmu \textrm{m}$ and an axial resolution of 5 $\upmu \textrm{m}$ in tissue. Three different acquisition areas were recorded:

- $\sim$ 3 $\times$ 3 $\textrm{mm}$ scan centered at the fovea consisting of 400 A-scans per B-scan and 400 consecutive B-scans
- $\sim$ 6.4 $\times$ 6.4 $\textrm{mm}$ scan centered at the fovea consisting of 512 A-scans per B-scan and 512 consecutive B-scans
- $\sim$ 6 $\times$ 6 $\textrm{mm}$ scan centered at the ONH consisting of 512 A-scans per B-scan and 512 consecutive B-scans

For each OCTA scan acquisition area the different retinal plexus projections were defined according to the approach presented by Campbell et al.^26^ based on pixel offsets of the retinal layer boundary segmentation of internal limiting membrane (ILM), nerve fibre layer (NFL), inner nuclear layer (INL) and outer plexiform layer (OPL) of the manufacturer’s software:

- NFLVP (nerve fibre layer vascular plexus): [ILM, NFL]
- SVP (superficial vascular plexus): [NFL, IPL-9]
- ICP (intermediate capillary plexus): [IPL-9, IPL+25]
- DCP (deep capillary plexus): [IPL+25, OPL+9]
- Retina (SVP + ICP + DCP): [ILM, OPL+9]

**Figure 1:**
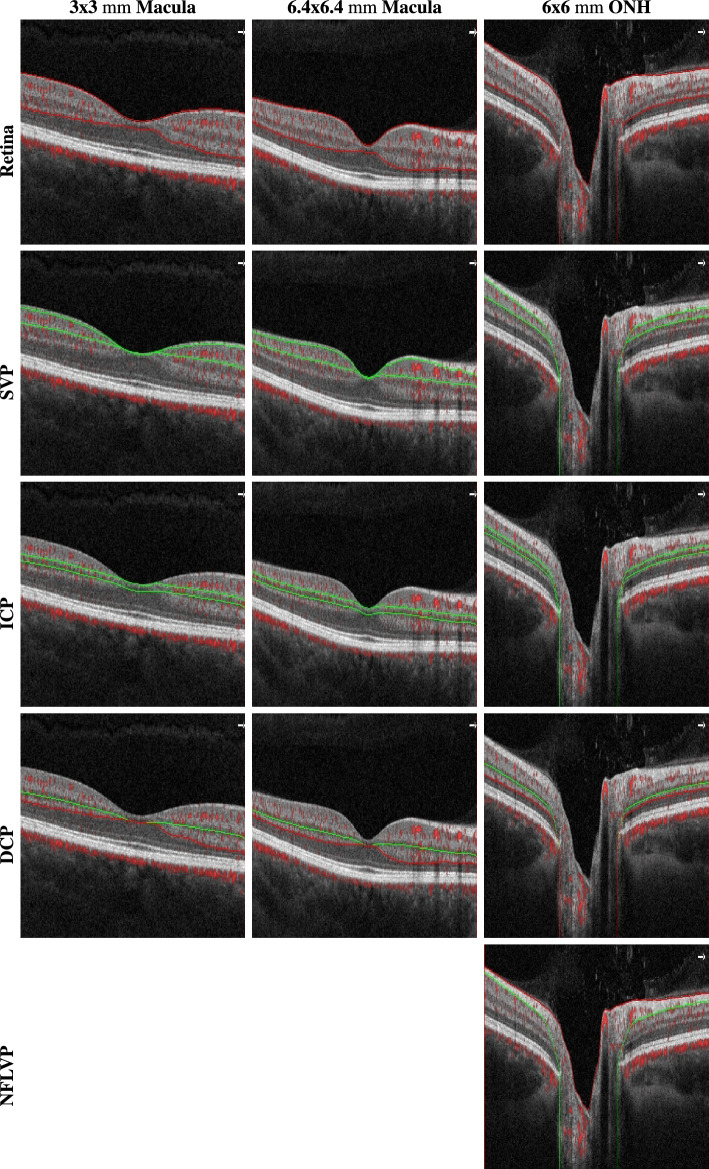
Visual impression of the projection boundaries of the different plexuses in the different acquisition areas from a right eye of a healthy control. The upper and lower boundaries are indicated in red or green.

**Figure 2:**
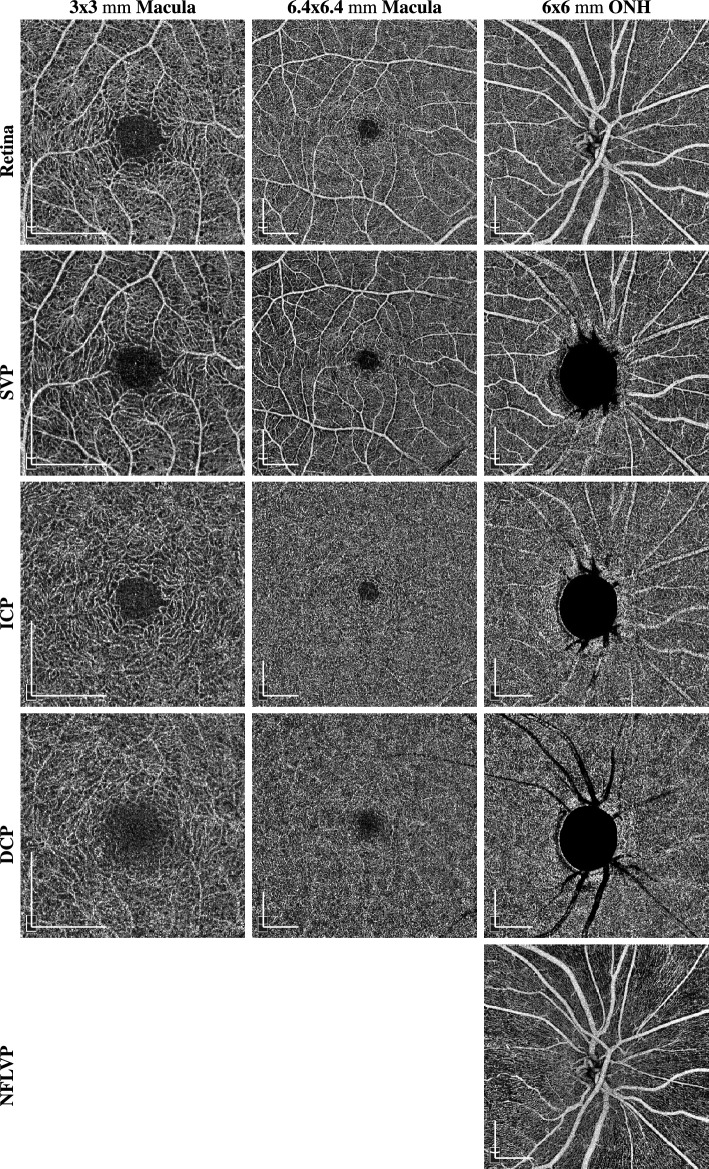
Visual impression of the appearance of the different plexuses in the different acquisition areas from a right eye of a healthy control. The scale bars in the lower left corners indicate 1$\textrm{mm}$.

SVP, ICP, DCP and Retina projections were computed by the manufacturer’s software for all acquisition areas. The NFL plexus was only generated for the 6 $\times$ 6 $\textrm{mm}$ ONH scans, since this capillary plexus is barely visible in the macular region. A depiction of the layer boundaries used for the plexus projection is provided in Fig. 1, while a visual impression of the appearance of the different plexuses in the different acquisition areas is provided in Fig. 2.

**Figure 3:**
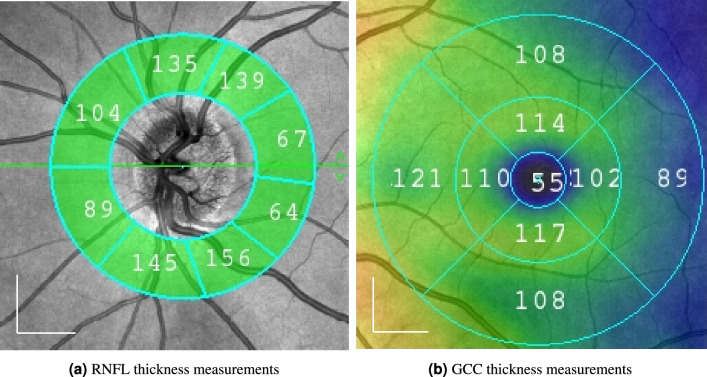
Depiction of the RNFL and GCC thickness parameters. For the RNFL thickness, 8 values were extracted from the 6 $\times$ 6 $\textrm{mm}$ ONH scans in a ring around the ONH, while for the GCC thickness 8 values were obtained from the 6.4 $\times$ 6.4 $\textrm{mm}$ macula scans from the inner and outer ring of the ETDRS grid. The scale bars in the lower left corners indicate 1$\textrm{mm}$.

When acquiring OCTA measurements, the SOLIX fullrange device (Optovue Inc, Fremont, California, USA) also gives access to the co-registered OCT measurements. Consequently, commonly used biomarkers for glaucoma diagnosis were extracted from the manufacturer’s software as well. One set of biomarkers were the ganglion cell complex (GCC) thickness. Here, 8 values were obtained from the 6.4 $\times$ 6.4 $\textrm{mm}$ macula scans from the inner and outer ring of the ETDRS grid. The second set of biomarkers were the retinal nerve fibre layer (RNFL) thickness. Again, 8 values were extracted from the 6 $\times$ 6 $\textrm{mm}$ ONH scans in a ring around the ONH. A visual example for the RNFL and GCC thickness parameter is fiven in Fig. 3. The third set of biomarkers contained three disc parameters, namely the disc area, the rim area and the cup area. The disc area is defined as the area inside the disc margin which is automatically detected based at the Bruch’s Membrane Opening (BMO). The rim and the cup are then measured within the BMO plane: the area above the BMO plane is rim, while the portion below the plane is cup. An overview of these parameters for the healthy control group and the glaucoma patients is given in the supplementary material S1.

All images were visually reviewed by ophthalmology experts who excluded images that they considered to be of insufficient quality for use in clinical routine.

The study was performed in accordance with the tenets of the Declaration of Helsinki and was approved by the local ethics committee of the University of Erlangen (3458, 295_20 B). Informed written consent was obtained from each participant.

This data was split into 60% training set, 20% validation set and 20% test set, with all eyes from one patient belonging exclusively to only one set. This leads to a distribution of 66/21/21 patients in the training/validation/test set.

### Experiments

In order to examine the impact of the acquisition area on the performance of deep learning-based approaches, we trained 13 CNNs on the different regions and plexuses displayed in Fig. 2. We chose the same hyperparameters that have worked best in our previous work^23^, namely a DenseNet161. Since the task to solve was the binary classification problem of distinguishing between glaucoma patients and healthy controls, binary cross-entropy was chosen as loss function. The networks were trained using stochastic gradient descent (SGD) with a momentum of 0.9, a learning rate of 0.001 and a batch size of 16. Moreover, the mean and standard deviation of the pixel intensities of all images have been calculated before the training and used to normalize the input images to have zero mean and a standard deviation of one. Class weights were assigned to be inversely proportional to the total amount of images per class in order to deal with the class imbalance inherent to the dataset.

For comparison, we also trained support vector machines with the VD as features for the same 13 regions and plexuses. Again the VD was computed as in our previous work^23^ by first matching the histogram of the input image to the one of a pre-selected scan. Afterwards the images were contrast enhanced using contrast limited adaptive histogram equalization (CLAHE) followed by a Frangi vesselness filter^27^ to highlight the vessel structures. In order to obtain a binary vessel map, hysteresis thresholding was applied. The VD was then computed as the fraction of vessel pixel to all pixel in the image.

Since biomarkers obtained from OCT scans are more established in the clinical routine in diagnosing and tracking progress of the disease, we also wanted to examine the performance of these biomarkers in comparison to our deep learning-based approach using OCTA. One set of biomarkers were the 8 values obtained from the GCC thickness maps. The second set of biomarkers were the 8 values obtained from the RNFL thickness maps. The third set of biomarkers contained three disc parameters, namely the disc area, the rim area and the cup area. For each of these three sets, SMVs were trained as well to distinguish between the glaucoma patients and the healthy controls.

In order to evaluate the performance of the different approaches the area under receiver operating characteristics (AUROC) was chosen as metric. Moreover 10 runs were carried out were in each run 20% of the data was randomly assigned to the test set and 20% of the data was randomly assigned to the validation set. The remaining 60% form the training set. It was taken care that all eyes from one patients exclusively belonged to only one of the sets.

A statistical analysis of the AUROC values of the 10 runs was performed in order to investigate which of the results are statistically significant different. This was done using an analysis of variance (ANOVA) model and Bonferroni correction was employed to account for multiple comparisons. The statistical analysis was done using SPSS version 28 (IBM Corp. Released 2021. IBM SPSS Statistics for Windows, Version 28.0. Armonk, NY: IBM Corp.) and a p-value less than 0.05 was considered statistically significant.

## Results and discussion

**Table 2:** Mean ± standard deviation of the AUROC values of the 10 runs for the evaluated CNNs, the SVM using the VD as a feature and the SVMs using the OCT parameters as features.

		3 $\times$ 3 $\textrm{mm}$ Macula	6.4 $\times$ 6.4 $\textrm{mm}$ Macula	6 $\times$ 6 $\textrm{mm}$ ONH
CNN	Retina	0.847 ± 0.070	0.870 ± 0.066	0.910 ± 0.046
	SVP	0.854 ± 0.079	0.888 ± 0.079	0.929 ± 0.040
	ICP	0.812 ± 0.075	0.858 ± 0.047	0.907 ± 0.046
	DCP	0.812 ± 0.090	0.869 ± 0.050	0.931 ± 0.051
	NFLVP	–	–	0.924 ± 0.034
VD	Retina	0.754 ± 0.023	0.728 ± 0.020	0.775 ± 0.050
	SVP	0.769 ± 0.029	0.737 ± 0.030	0.800 ± 0.042
	ICP	0.790 ± 0.023	0.773 ± 0.023	0.786 ± 0.030
	DCP	0.778 ± 0.009	0.758 ± 0.009	0.609 ± 0.225
	NFLVP	–	–	0.809 ± 0.076
OCT	GCC	–	0.758 ± 0.061	–
	RNFL	–	–	0.871 ± 0.057
	Disc	–	–	0.911 ± 0.043

**Figure 4:**
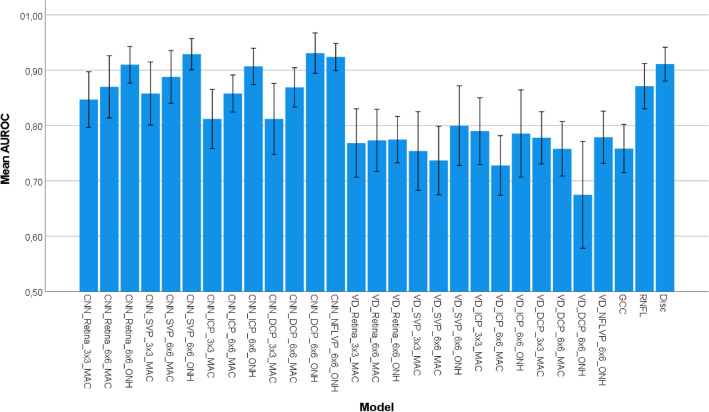
Mean AUROC values with their 95% confidence intervals for the different methods, acquisition areas and plexuses over the 10 runs.

The mean and standard deviation AUROC values of the ten runs for the three different experiments for the different acquisition areas and plexuses can be found in Table 2. A plot with the mean AUROC values and their 95% confidence intervals for the different methods, acquisition areas and plexuses over the 10 folds is shown in Fig. 4.

When looking at the results obtained by the CNN we observe that all show the same tendency for the 3 $\times$ 3 $\textrm{mm}$ macula scans as in our previous work^23^, with the SVP performing best, followed by the retina projection and the ICP and DCP performing similarly. However, the obtained AUROC values are lower than before. A reason might be, that the dataset in this work is different in regard to the patient cohort and the device. This dataset might contain more patients with earlier stages of glaucoma, making the distinction between the glaucoma patients and the healthy controls more difficult. Moreover, the resolution of the 3 $\times$ 3 $\textrm{mm}$ macula scans of the OCT device in this work is smaller (400 $\times$ 400 pixels) than the one in our previous work (512 $\times$ 512 pixels). This might also influence the visibility of small capillaries.

However, for the results obtained on our data the CNNs show the best performance on the 6 $\times$ 6 $\textrm{mm}$ ONH, followed by the 6.4 $\times$ 6.4 $\textrm{mm}$ macular scans and the 3 $\times$ 3 $\textrm{mm}$ macular scans, which is in line with results reported in the literature for the VD^14–17^. Moreover it shows that for the macular scans the SVP and the Retina projections seem to have the highest discriminative power while for the ONH scans the SVP, the RNFL, and the DCP are showing high AUROC values.

When examining the results obtained by the SVMs trained on the VD they do not provide a conclusive answer as in the case of the CNNs. The best performance here is also obtained using the 6 $\times$ 6 $\textrm{mm}$ ONH, however on our data the larger 6.4 $\times$ 6.4 $\textrm{mm}$ macular scans are not performing better than the smaller 3 $\times$ 3 $\textrm{mm}$ ones. In the literature, groups report AUROC values for the whole image VD ranging from 0.651 to 0.73 for the 3 $\times$ 3 $\textrm{mm}$ scans^14–16^, from 0.74 to 0.961 for the 6 $\times$ 6 $\textrm{mm}$ macular scans^13,17,28^, from 0.79 to 0.94 for 4.5 $\times$ 4.5 $\textrm{mm}$ ONH^14,16,29–31^ and 0.671 for the 6 $\times$ 6 $\textrm{mm}$ ONH scans^19^. However, none of these papers compared the smaller to the larger field of view on the same dataset as we did in our work, so the results are not comparable. Even when looking at the range of results from the same acquisition areas it spans a wide range. One reason therefore might be that the patient cohorts differ in the amount of included glaucoma patients and/or in the distribution of glaucoma patients across disease severity. Another reason might be that groups are using different OCT devices which might differ in the resolution of the acquisition areas and/or also in the post-processing. Having the same acquisition area with a lower resolution might lead to the problem that small structures are not visible anymore. The post-processing of the manufacturers’ devices leads to a different input to the vessel density computation such that the same algorithm can also lead to different results from the same eye of one patient on two different devices. Moreover the computation of the vessel density needs a segmented vessel map. This binarization can be performed in many different ways, or may not even by known if the vessel density is computed directly on the OCT device, and depends very sensitively on the choice of parameters in order to get the best results. All of this can be reasons for the wide range of reported results and renders them incomparable if different acquisition areas are not tested on the same dataset from the same device with the same algorithms.

Although these problems of creating comparable results also exist to a certain extend when using CNNs, we can see on our dataset that for every combination of acquisition area and plexus the CNNs outperform the vessel density. So our deep learning approach has higher AUROC values and outperforms the traditional machine learning with handcrafted features for every acquisition area and plexus.

When looking at the results from the OCT measurements we can see that the GCC thickness is the least predictive biomarker followed by the RNFL thickness and the disc parameters. So here also the ONH parameters are performing better than the macular ones. These OCT biomarkers are used in the clinical routine for diagnosing and tracking disease progression. In contrast to that, OCTA is not commonly used for this task. However, our results show that the CNNs trained on the ONH scans show a better distinction than these traditional biomarkers. Consequently OCTA could be a useful addition to OCT in the clinical routine and research. However, we only used handcrafted features as OCT biomarkers. It is unclear as of yet whether even better results can be obtained by applying CNNs to this task. But this would increase computational complexity since most likely 3D data would be needed as input to the networks.

The statistical analysis revealed that for the CNNs there is no statistical difference between any combination of acquisition area and plexus. The SVMs trained on the VD are also statistically indistinguishable from each other. For the CNNs the 6 $\times$ 6 $\textrm{mm}$ ONH DCP setup was statistically significantly different in 14 out of 16 comparisons (except for the 6 $\times$ 6 $\textrm{mm}$ ONH OCT values). Moreover were the 6.4 $\times$ 6.4 $\textrm{mm}$ Macula SVP and the 6 $\times$ 6 $\textrm{mm}$ ONH NFLVP statistically significantly different in 13 out of 16 comparisons (except for 6 $\times$ 6 $\textrm{mm}$ Macula SVP of the VD and the 6 $\times$ 6 $\textrm{mm}$ ONH OCT values). For the SVMs trained on the VD the most interesting setup was the 6 $\times$ 6 $\textrm{mm}$ ONH DCP which was statistically significantly different in 15 out of 16 comparisons (except for the GCC thickness). The table with all p-values of the pairwise comparisons is given in the supplementary material S2.

**Figure 5:**
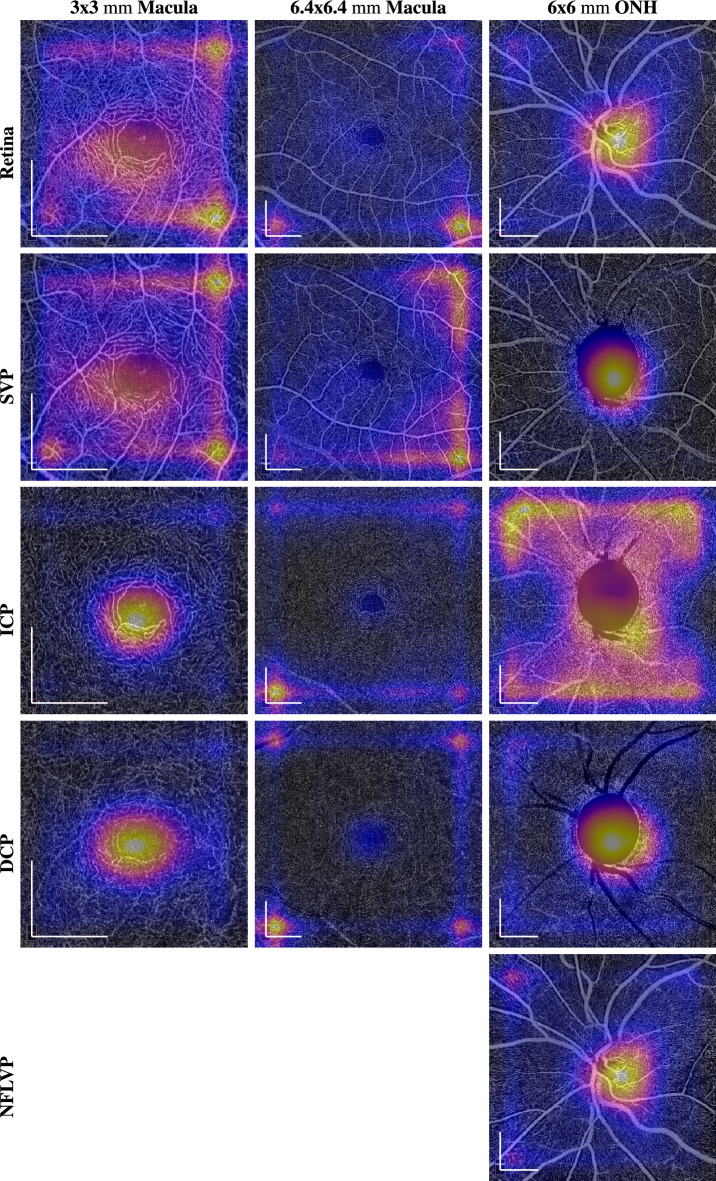
Averaged Grad-CAM heatmaps of correctly identified glaucoma patients of the test sets displayed on the en face OCTA projections of the right eye of a healthy subject for the different plexuses and acquisition areas. The heatmaps of left eyes were mirrored before averaging. The scale bars in the lower left corners indicate 1$\textrm{mm}$.

In order to investigate which regions are of special interest to the network in the different acquisition areas and plexuses, we computed the average Grad-CAM heatmaps of all images in the test sets and displayed them in Fig. 5 on the OCTA en face images of a healthy subject in order to get an idea of the underlying anatomical structures. One observation was that the highlighted areas at the borders and edges of the image all have a block-like appearance. We assume that this is an artifact from the Grad-CAM algorithm since the heatmaps are computed on a lower scale and are subsequently upsampled to be displayed on the original input images. For the 3 $\times$ 3 $\textrm{mm}$ macular scans the network mostly focuses on the edges on the image and to a smaller degree on the fovea for the SVP and Retina projection and soley on the area of the fovea for the ICP and DCP projection. The latter two perform similarly and worse, which might indicate that there is additional information in the corners of the projections. For the 6.4 $\times$ 6.4 $\textrm{mm}$ macular scans the networks seem to focus on the corners of the images again and to a lesser degree at the fovea for all plexuses. In addition, there is also a light highlight extending from the corners towards the center for the SVP and Retina projections. Since these two showed the best performance this might indicate that not only in the corners a pathologicaly induced change can be observed but rather already in those regions just outside the 3 $\times$ 3 $\textrm{mm}$ area. For the 6 $\times$ 6 $\textrm{mm}$ ONH scans, the focus mainly lies in the region of the optic disc with a slight focus also on the corners again (except for the ICP where also a heavy focus lies on the edges on corners but also has the worst performance). This indicates that the area around the optic disc carries the most information about glaucomatous change. One interesting observation is that even in the SVP, ICP and DCP where there is no information about the vasculature, the networks focus on this area. This black spot comes from the definition of the layer boundaries around the optic disc, which all stop at the optic disc border. Consequently no pixels are included from the region within the optic disc when computing the en face projection. This observation can lead to two different conclusions. On the one hand, it might be that especially the vasculature around the optic disc shows changes. On the other hand, we have seen that the optic disc parameters obtained from the OCT measurements were the best performing biomarkers. So information about the optic disc might be observeable in this region, even if only the shape of the optic disc is visible. In order to get to meaningful conclusions regarding this observation, further research is necessary. More examples of Grad-CAM from individual glaucoma patients are given in the supplementary material S3.

Patients with a peripapillary atrophy (PPA) and/or myopia were not excluded from this study. However, multiple studies found, that PPA and high myopia can influence the retinal microvasculature^32–34^. Consequently, this could have an influence on the results of our study. But patients with PPA and/or myopia were present in the glaucoma cohort as well as in the control cohort. Therefore, the groups are unbiased in this regard. Another interesting research direction would be to look at the different subtypes of glaucoma separately. In this work all glaucoma subtypes were treated equally to just belong to the classification group of glaucoma. However, different subtypes might be affected differently which might be embedded in the en face images. However, this would need much more patient data, since enough data for training, validation and testing is necessary for each of these subtypes. We hope that in the future we can acquire this data.

Moreover in this work we have only included the 6 $\times$ 6 $\textrm{mm}$ ONH scans into our evaluation while most vessel density studies used a 4.5 $\times$ 4.5 $\textrm{mm}$ region centered at the optic disc. However, since the networks seem to mostly focus on the area of the optic disc, which is visible independent of the field of view, we would assume that one can achieve similar results with this smaller scan size.

Images with insufficient quality for clinical use were excluded from the study by ophthalmology experts. Artifacts may still be present and the signal strength may not always be optimal, which can affect the segmentation of the retinal layers and thus the calculated metrics. However, this data represents a real-world dataset that is used in the clinical routine on which clinicians will work on. Consequently, the results of this study are representative for real-world applications and research.

## Summary and conclusion

We have trained CNNs on 13 different acquisition areas (3 $\times$ 3 $\textrm{mm}$ macular scans, 6.4 $\times$ 6.4 $\textrm{mm}$ macular scans and 6 $\times$ 6 $\textrm{mm}$ ONH scans) and plexuses (whole retina, SVP, ICP, DCP and NFLVP (only at ONH scans)) in order to examine the influence of them for the task of distinguishing glaucoma patients from healthy controls. The results show, that the OCTA ONH scans are especially predicitive for this task. Moreover, we could show, that the deep learning-based approach outperforms the most commonly used biomarker for OCTA scans, the vessel density, on every acqusition area and plexus. Moreover when comparing the results to the ones obtained from OCT measurements, that are used in the clinical routine for diagnosing and tracking disease progression, the ONH OCTA scans perform slightly better. Consequently OCTA ONH scans might be a useful addition to OCT for studying glaucoma. When looking at the Grad-CAM heatmaps, the networks seem to focus mostly on the region of the optic disc. However, further research is necessary to uncover the pathological mechanism in this area.

### Supplementary Information

Supplementary Information.

## Data Availability

The dataset analysed during the current study is not publicly available due to general data privacy regulations but is available from the corresponding author on reasonable request.
