# Supplementary material for: Impact of acquisition area on deep-learning-based glaucoma detection in different plexuses in OCTA
Source: Sci Rep. 2024 Sep 2;14:20414. doi: 10.1038/s41598-024-71235-3 (PMC11369139; doi:10.1038/s41598-024-71235-3)
Supplement: Supplementary file 1 — Supplementary Information. [file 41598_2024_71235_MOESM1_ESM.pdf]

	Healthy Cohort	Glaucoma Cohort
GCC S i	114.61 ± 8.14	101.02 ± 19.46
GCC N i	112.75 ± 8.63	99.80 ± 17.23
GCC I i	115.59 ± 8.63	97.10 ± 20.81
GCC T i	102.83 ± 8.14	88.23 ± 17.50
GCC S o	100.06 ± 7.50	85.02 ± 19.59
GCC N o	116.85 ± 8.44	100.62 ± 17.44
GCC I o	99.89 ± 8.50	81.01 ± 16.84
GCC T o	85.52 ± 6.75	72.41 ± 12.30
RNFL SN	107.20 ± 22.50	82.48 ± 28.32
RNFL NS	85.23 ± 15.16	68.88 ± 28.30
RNFL NI	66.39 ± 15.45	56.61 ± 16.30
RNFL IN	113.25 ± 23.45	76.74 ± 28.07
RNFL TI	134.94 ± 20.74	88.96 ± 40.11
RNFL TI	61.56 ± 14.19	53.07 ± 14.50
RNFL TS	66.24 ± 13.66	56.57 ± 13.46
RNFL ST	123.58 ± 24.38	84.78 ± 30.38
Disc Area	1.82 ± 0.62	1.81 ± 0.57
Rim Area	1.44 ± 0.58	0.70 ± 0.50
Cup Area	0.38 ± 0.30	1.12 ± 0.63
VD 3x3 mm Macula Retina	0.371 ± 0.027	0.33 ± 0.049
VD 3x3 mm Macula SVP	0.394 ± 0.025	0.0.354 ± 0.041
VD 3x3 mm Macula ICP	0.250 ± 0.014	0.236 ± 0.021
VD 3x3 mm Macula DCP	0.234 ± 0.018	0.226 ± 0.018
VD 6.4x6.4 mm Macula Retina	0.359 ± 0.020	0.341 ± 0.029
VD 6.4x6.4 mm Macula SVP	0.363 ± 0.017	0.346 ± 0.026
VD 6.4x6.4 mm Macula ICP	0.274 ± 0.013	0.271 ± 0.019
VD 6.4x6.4 mm Macula DCP	0.277 ± 0.014	0.275 ± 0.015
VD 6x6 mm ONH Retina	0.401 ± 0.027	0.364 ± 0.041
VD 6x6 mm ONH SVP	0.388 ± 0.019	0.360 ± 0.021
VD 6x6 mm ONH ICP	0.303 ± 0.018	0.283 ± 0.026
VD 6x6 mm ONH DCP	0.315 ± 0.013	0.305 ± 0.026
VD 6x6 mm ONH NFLVP	0.355 ± 0.019	0.337 ± 0.019

S1: Mean ± standard deviation of the GCC and RFNL thicknesses, disc and vessel density parameters. S = superior, N = nasal, I = inferior, T = temporal, i = inner, o = outer.

			CNN												
			3x3 mm Macula				6.4x6.4 mm Macula				6x6 mm ONH				
			Retina	SVP	ICP	DCP	Retina	SVP	ICP	DCP	Retina	SVP	ICP	DCP	NFLVP
CNN	3x3 mm Macula	Retina													
		SVP	1.000												
		ICP	1.000	1.000											
		DCP	1.000	1.000	1.000										
	6.4x6.4 mm Macula	Retina	1.000	1.000	1.000	1.000									
		SVP	1.000	1.000	1.000	1.000	1.000								
		ICP	1.000	1.000	1.000	1.000	1.000	0.202							
		DCP	1.000	1.000	1.000	1.000	1.000	1.000	1.000						
	6x6 mm ONH	Retina	1.000	1.000	1.000	1.000	1.000	1.000	1.000	1.000					
		SVP	1.000	1.000	1.000	1.000	1.000	0.202	1.000	1.000	1.000				
		ICP	1.000	1.000	1.000	1.000	1.000	1.000	1.000	1.000	1.000	1.000			
		DCP	1.000	1.000	1.000	1.000	1.000	1.000	0.163	1.000	1.000	0.163	1.000		
		NFLVP	1.000	1.000	1.000	1.000	1.000	1.000	0.345	1.000	1.000	0.345	1.000	1.000	
VD	3x3 mm Macula	Retina	1.000	0.993	**0.011**	1.000	0.153	**0.001**	1.000	1.000	**0.017**	1.000	1.000	**0.001**	**0.002**
		SVP	1.000	1.000	**0.021**	1.000	0.258	**0.002**	1.000	1.000	**0.030**	1.000	1.000	**0.001**	**0.003**
		ICP	1.000	1.000	**0.025**	1.000	0.302	**0.002**	1.000	1.000	**0.035**	1.000	1.000	**0.002**	**0.004**
		DCP	1.000	0.227	**0.002**	0.784	**0.029**	**0.000**	1.000	0.784	**0.003**	1.000	0.253	**0.000**	**0.000**
	6.4x6.4 mm Macula	Retina	0.426	**0.033**	**0.000**	0.131	**0.003**	**0.000**	1.000	0.131	**0.000**	1.000	**0.037**	**0.000**	**0.000**
		SVP	1.000	1.000	0.418	1.000	1.000	0.051	1.000	1.000	0.568	1.000	1.000	**0.040**	0.091
		ICP	1.000	1.000	0.146	1.000	1.000	**0.016**	1.000	1.000	0.204	1.000	1.000	**0.012**	**0.029**
		DCP	0.162	**0.011**	**0.000**	**0.046**	**0.001**	**0.000**	1.000	**0.046**	**0.000**	1.000	**0.012**	**0.000**	**0.000**
	6x6 mm ONH	Retina	1.000	1.000	0.089	1.000	0.918	**0.009**	1.000	1.000	0.125	1.000	1.000	**0.007**	**0.017**
		SVP	1.000	1.000	**0.036**	1.000	0.421	**0.003**	1.000	1.000	0.052	1.000	1.000	**0.003**	**0.006**
		ICP	1.000	0.345	**0.003**	1.000	**0.046**	**0.000**	1.000	1.000	**0.004**	1.000	0.384	**0.000**	**0.000**
		DCP	**0.000**	**0.000**	**0.000**	**0.000**	**0.000**	**0.000**	**0.019**	**0.000**	**0.000**	**0.019**	**0.000**	**0.000**	**0.000**
		NFLVP	1.000	1.000	**0.040**	1.000	0.463	**0.004**	1.000	1.000	0.058	1.000	1.000	**0.003**	**0.007**
OCT	6.4x6.4 mm Macula	GCC	1.000	0.363	**0.003**	1.000	**0.049**	**0.000**	1.000	1.000	**0.005**	1.000	0.403	**0.000**	**0.000**
	6x6 mm ONH	RNFL	1.000	1.000	1.000	1.000	1.000	1.000	1.000	1.000	1.000	1.000	1.000	1.000	1.000
		Disc	1.000	1.000	1.000	1.000	1.000	1.000	1.000	1.000	1.000	1.000	1.000	1.000	1.000

			VD												
			3x3 mm Macula				6.4x6.4 mm Macula				6x6 mm ONH				
			Retina	SVP	ICP	DCP	Retina	SVP	ICP	DCP	Retina	SVP	ICP	DCP	NFLVP
CNN	3x3 mm Macula	Retina													
		SVP													
		ICP													
		DCP													
	6.4x6.4 mm Macula	Retina													
		SVP													
		ICP													
		DCP													
	6x6 mm ONH	Retina													
		SVP													
		ICP													
		DCP													
		NFLVP													
VD	3x3 mm Macula	Retina													
		SVP	1.000												
		ICP	1.000	1.000											
		DCP	1.000	1.000	1.000										
	6.4x6.4 mm Macula	Retina	1.000	1.000	1.000	1.000									
		SVP	1.000	1.000	1.000	1.000	1.000								
		ICP	1.000	1.000	1.000	1.000	1.000	1.000							
		DCP	1.000	1.000	1.000	1.000	1.000	1.000	1.000						
	6x6 mm ONH	Retina	1.000	1.000	1.000	1.000	1.000	1.000	1.000	1.000					
		SVP	1.000	1.000	1.000	1.000	1.000	1.000	1.000	1.000	1.000				
		ICP	1.000	1.000	1.000	1.000	1.000	1.000	1.000	1.000	1.000	1.000			
		DCP	1.000	1.000	1.000	1.000	1.000	1.000	0.243	1.000	0.387	0.848	1.000		
		NFLVP	1.000	1.000	1.000	1.000	1.000	0.082	1.000	1.000	1.000	1.000	0.848	0.733	
OCT	6.4x6.4 mm Macula	GCC	1.000	1.000	1.000	1.000	1.000	1.000	1.000	1.000	1.000	1.000	1.000	1.000	1.000
	6x6 mm ONH	RNFL	0.885	1.000	1.000	0.200	**0.028**	1.000	1.000	**0.009**	1.000	1.000	1.000	**0.000**	1.000
		Disc	**0.010**	**0.018**	**0.021**	**0.001**	**0.000**	0.369	0.128	**0.000**	0.078	**0.031**	**0.002**	**0.000**	**0.035**

			OCT		
			6.4x6.4 mm Macula GCC	6x6 mm ONH RNFL	6x6 mm ONH Disc
CNN	3x3 mm Macula	Retina			
		SVP			
		ICP			
		DCP			
	6.4x6.4 mm Macula	Retina			
		SVP			
		ICP			
		DCP			
	6x6 mm ONH	Retina			
		SVP			
		ICP			
		DCP			
		NFLVP			
VD	3x3 mm Macula	Retina			
		SVP			
		ICP			
		DCP			
	6.4x6.4 mm Macula	Retina			
		SVP			
		ICP			
		DCP			
	6x6 mm ONH	Retina			
		SVP			
		ICP			
		DCP			
		NFLVP			
OCT	6.4x6.4 mm Macula	GCC			
	6x6 mm ONH	RNFL	0.321		
		Disc	**0.003**	1.000	

S2: P-values for the statistical analysis of the results of the different methods, acquisition areas and plexuses. Bonferroni adjustment was used to account for multiple comparisons. Statistically significant are highlighted in bold.

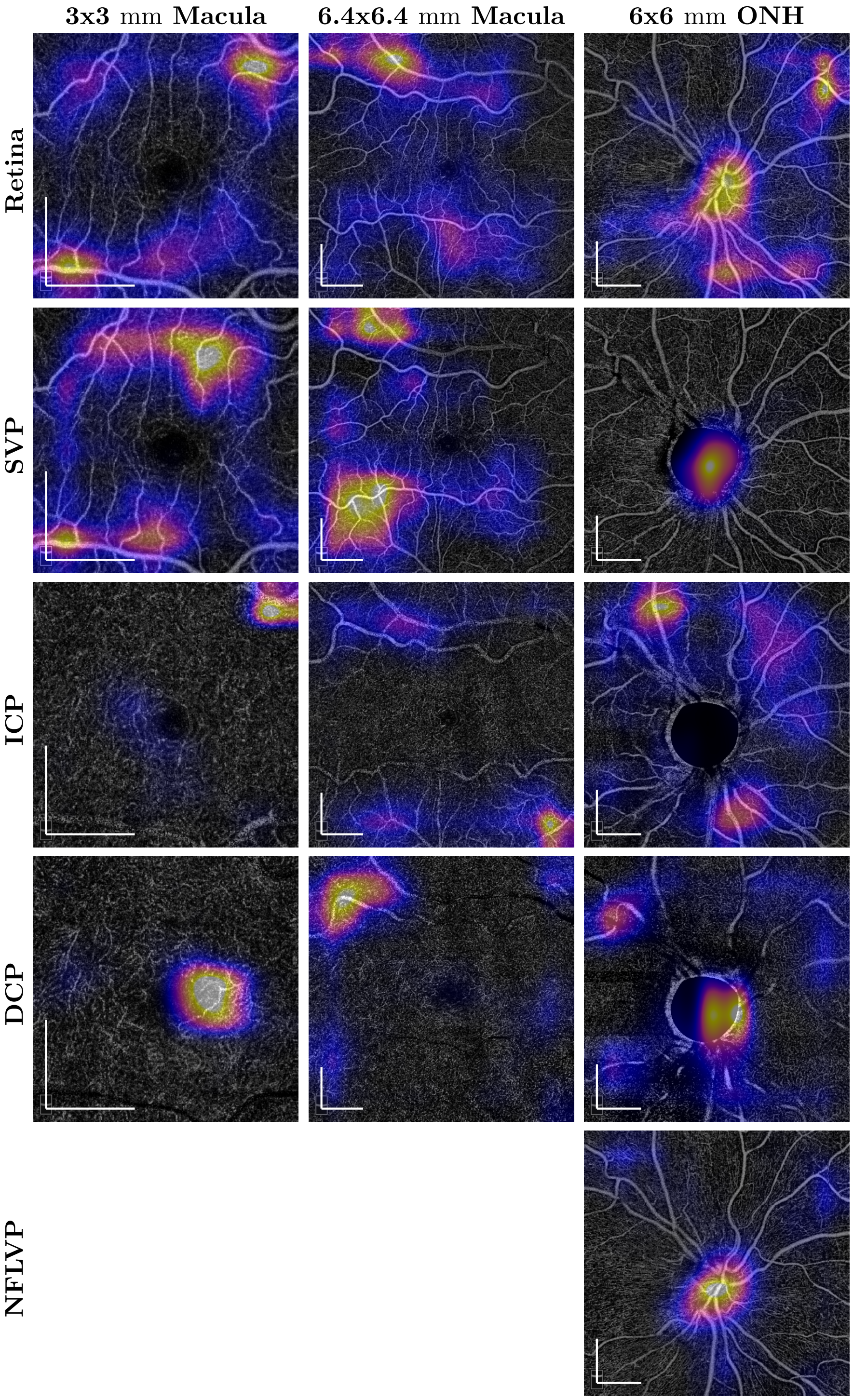
3x3 mm Macula
6.4x6.4 mm Macula
6x6 mm ONH
Retina
SVP
ICP
DCP
NFLVP

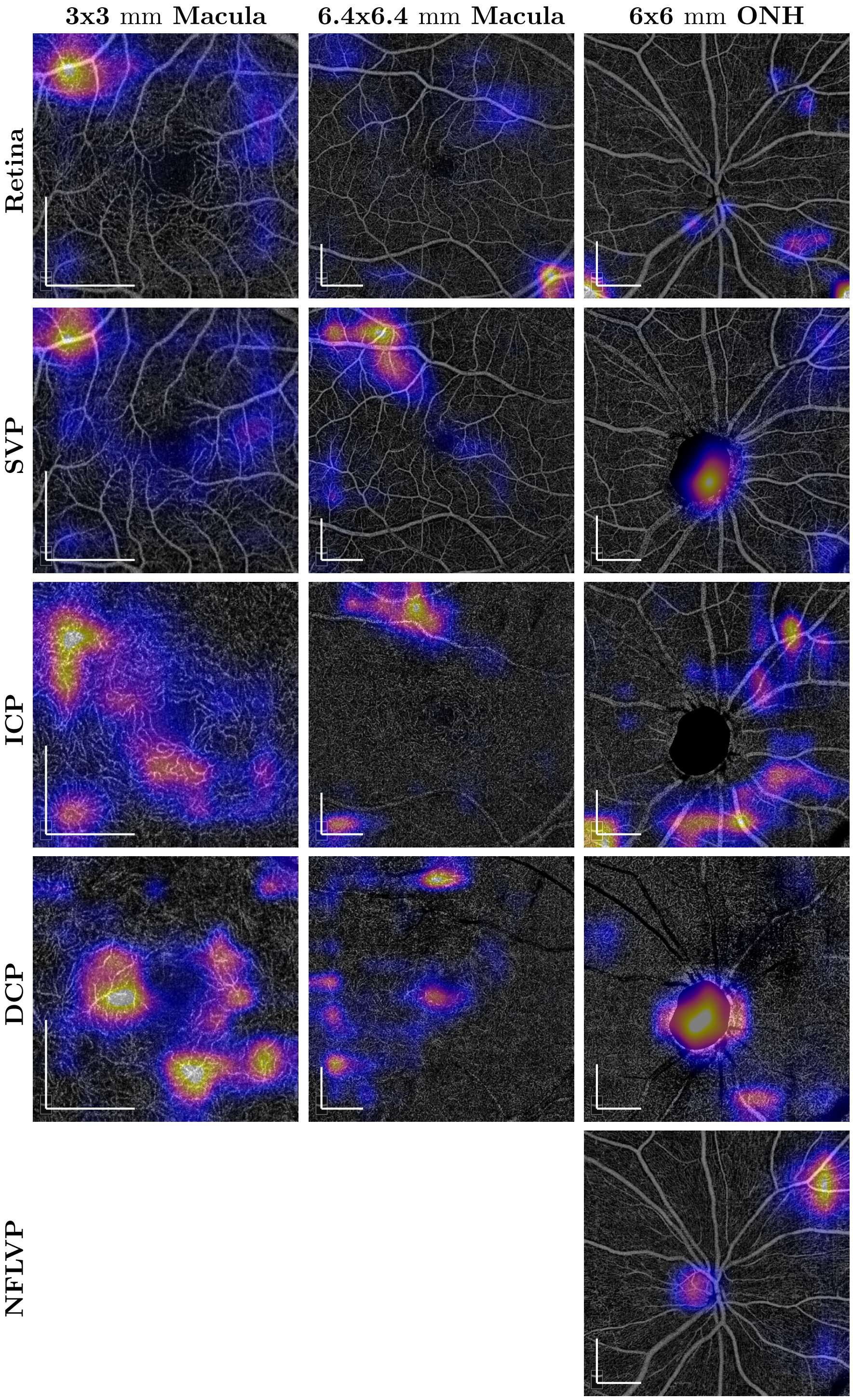
3x3 mm Macula
6.4x6.4 mm Macula
6x6 mm ONH
Retina
SVP
ICP
DCP
NFLVP

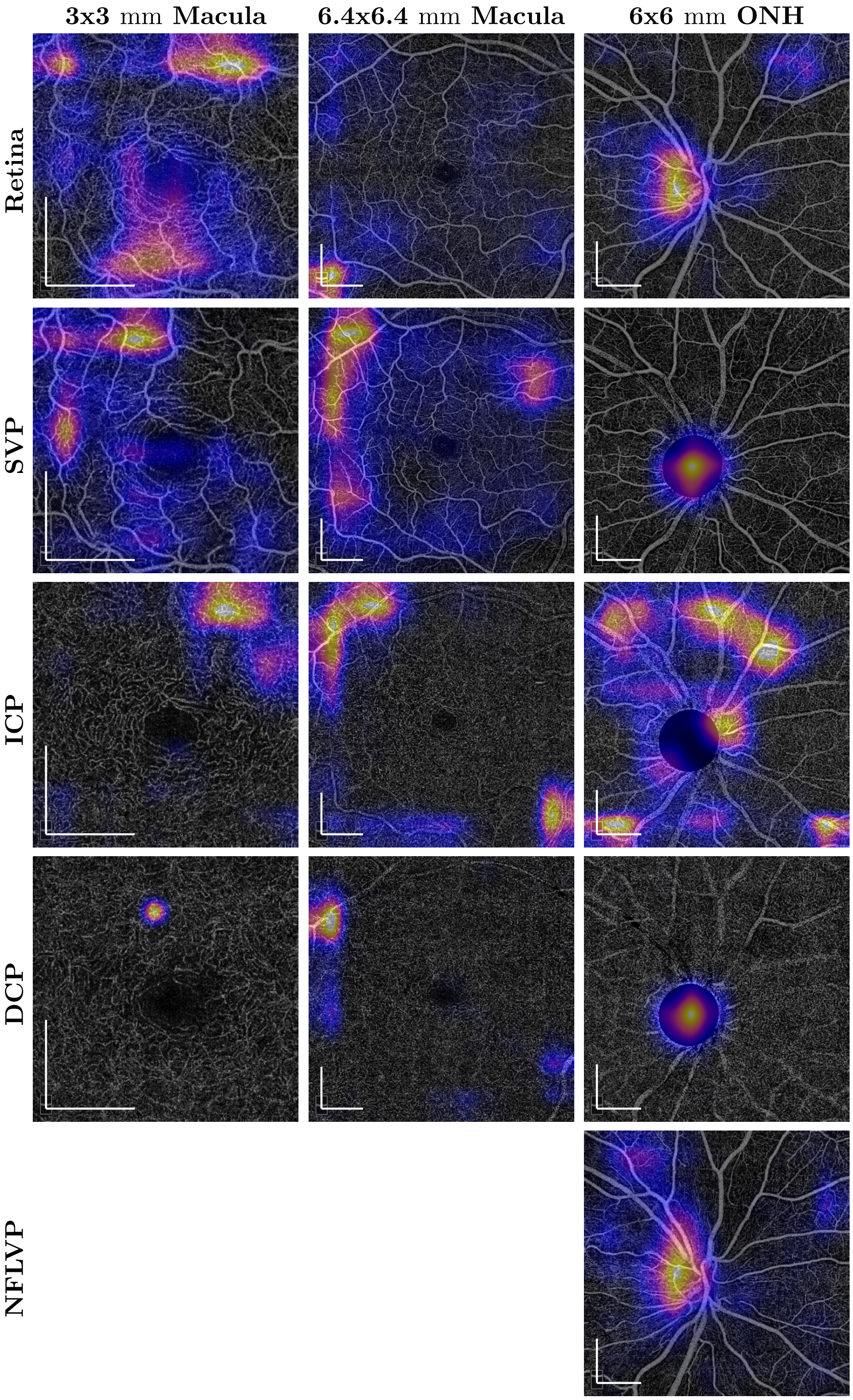

3x3 mm Macula
6.4x6.4 mm Macula
6x6 mm ONH
Retina
SVP
ICP
DCP
NFLVP

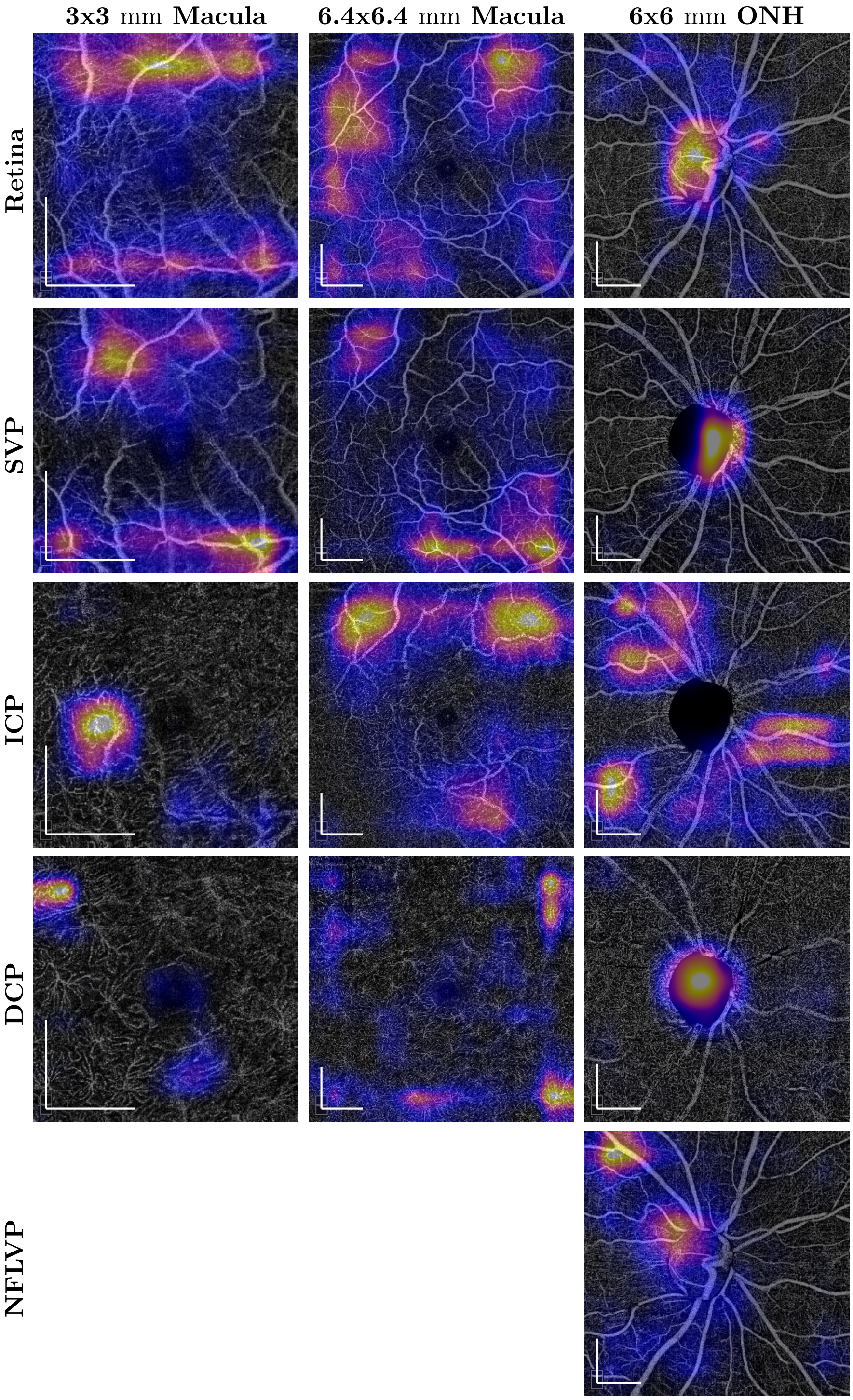
3x3 mm Macula
6.4x6.4 mm Macula
6x6 mm ONH
Retina
SVP
ICP
DCP
NFLVP

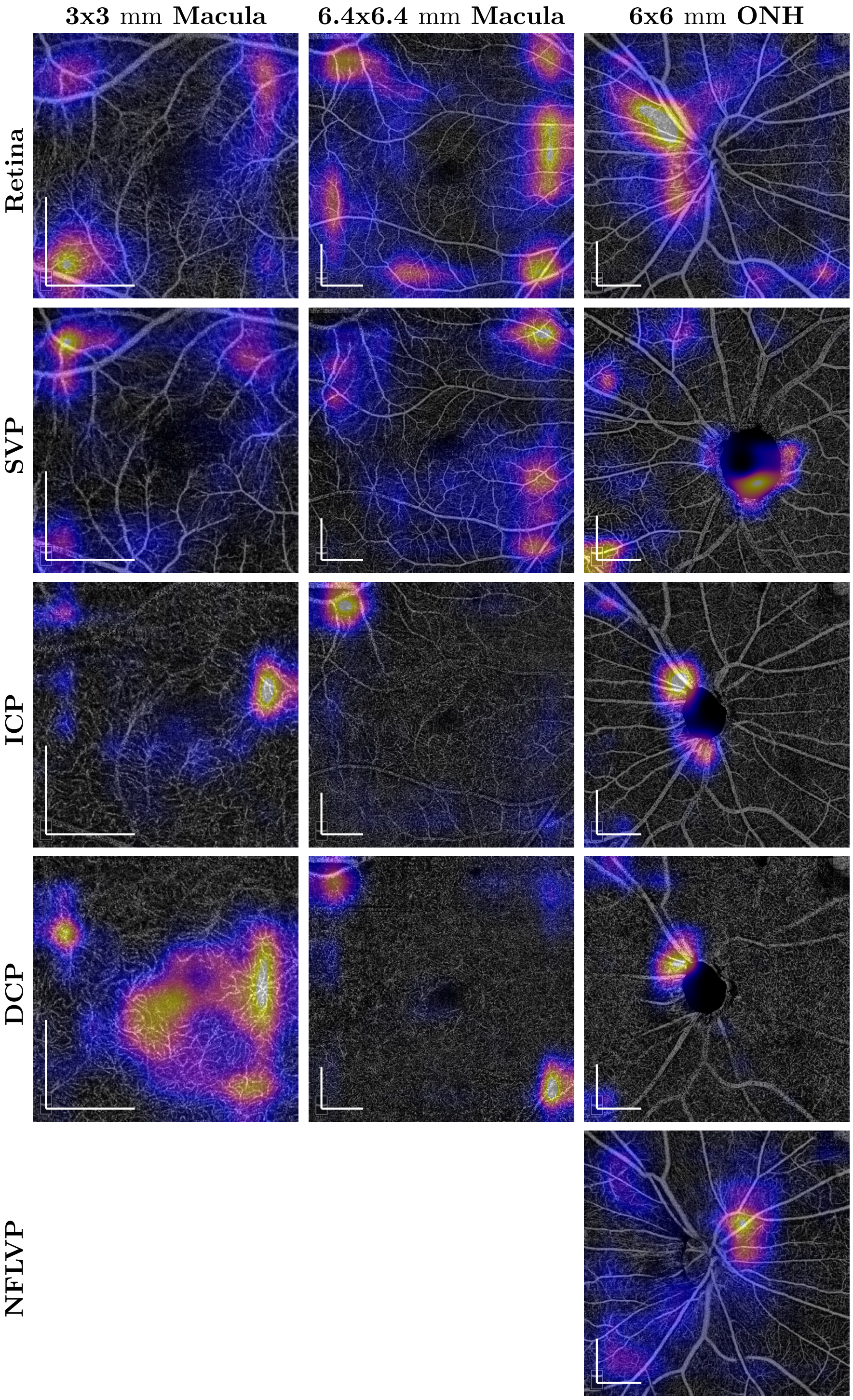

3x3 mm Macula
6.4x6.4 mm Macula
6x6 mm ONH
Retina
SVP
ICP
DCP
NFLVP

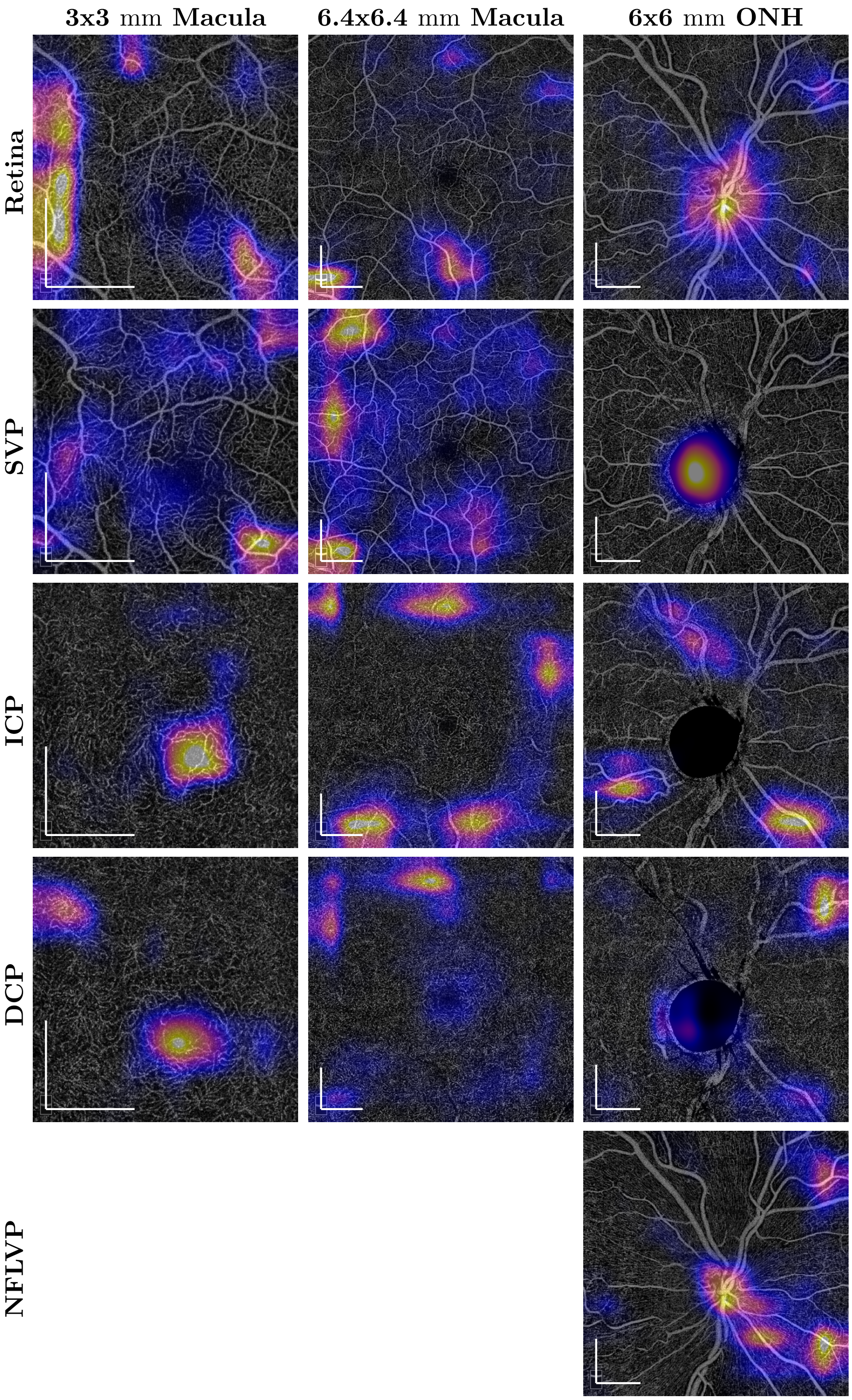

S3: Visual impression of the Grad-CAM map appearances of the different plexuses in the different acquisition areas from individual glaucoma patients. The scale bars in the lower left corners indicate 1mm.
